# Effect of exercise and diet intervention in NAFLD and NASH via *GAB2* methylation

**DOI:** 10.1186/s13578-021-00701-6

**Published:** 2021-11-04

**Authors:** Na Wu, Fan Yuan, Siran Yue, Fengyan Jiang, Decheng Ren, Liangjie Liu, Yan Bi, Zhenming Guo, Lei Ji, Ke Han, Xiao Yang, Mofan Feng, Kai Su, Fengping Yang, Xi Wu, Qing Lu, Xingwang Li, Ruirui Wang, Baocheng Liu, Shenglong Le, Yi Shi, Guang He

**Affiliations:** 1 Bio-X Institutes, Key Laboratory for the Genetics of Developmental and Neuropsychiatric Disorders, Shanghai Jiao Tong University, 1954 Huashan Road, Shanghai, 200030 China; 2 Shanghai Innovation Center of Traditional Chinese Medicine Health Service, Shanghai University of Traditional Chinese Medicine, Shanghai, China; 3 Shanghai Key Laboratory of Psychotic Disorders, and Brain Science and Technology Research Center, Shanghai Jiao Tong University, Shanghai, China; 4 Exercise Translational Medicine Center, Shanghai Center for Systems Biomedicine, Shanghai Jiao Tong University, Shanghai, China

**Keywords:** NAFLD, NASH, Exercise intervention, Diet intervention, DNA methylation, *GAB2*

## Abstract

**Background:**

Nonalcoholic fatty liver disease (NAFLD) is a disorder that extends from simple hepatic steatosis to nonalcoholic steatohepatitis (NASH), which is effectively alleviated by lifestyle intervention. Nevertheless, DNA methylation mechanism underling the effect of environmental factors on NAFLD and NASH is still obscure. The aim of this study was to investigate the effect of exercise and diet intervention in NAFLD and NASH via DNA methylation of *GAB2*.

**Methods:**

Methylation of genomic DNA in human NAFLD was quantified using Infinium Methylation EPIC BeadChip assay after exercise (Ex), low carbohydrate diet (LCD) and exercise plus low carbohydrate diet (ELCD) intervention. The output Idat files were processed using ChAMP package. False discovery rate on genome-wide analysis of DNA methylation (q < 0.05), and cytosine-guanine dinucleotides (CpGs) which are located in promoters were used for subsequent analysis (|Δβ|≥ 0.1). K-means clustering was used to cluster differentially methylated genes according to 3D genome information from Human embryonic stem cell. To quantify DNA methylation and mRNA expression of *GRB2* associated binding protein 2 (*GAB2*) in NASH mice after Ex, low fat diet (LFD) and exercise plus low fat diet (ELFD), MassARRAY EpiTYPER and quantitative reverse transcription polymerase chain reaction were used.

**Results:**

Both LCD and ELCD intervention on human NAFLD can induce same DNA methylation alterations at critical genes in blood, e.g., *GAB2*, which was also validated in liver and adipose of NASH mice after LFD and ELFD intervention. Moreover, methylation of CpG units (i.e., CpG_10.11.12) inversely correlated with mRNA expression *GAB2* in adipose tissue of NASH mice after ELFD intervention.

**Conclusions:**

We highlighted the susceptibility of DNA methylation in *GAB2* to ELFD intervention, through which exercise and diet can protect against the progression of NAFLD and NASH on the genome level, and demonstrated that the DNA methylation variation in blood could mirror epigenetic signatures in target tissues of important biological function, i.e., liver and adipose tissue.

*Trial* *registration* International Standard Randomized Controlled Trial Number Register (ISRCTN 42622771)

**Supplementary Information:**

The online version contains supplementary material available at 10.1186/s13578-021-00701-6.

## Introduction

Nonalcoholic fatty liver disease (NAFLD) and nonalcoholic steatohepatitis (NASH) can increase the risk of hepatocellular carcinoma, type 2 diabetes and cardiovascular diseases [2–4], which are known to be the most common causes of mortality worldwide [5]. It was estimated that the NAFLD and NASH population in China would increase by 26.2% and 48% by 2030, respectively [6].

Previous studies have shown that exercise and diet play important roles in the treatment of NAFLD and NASH [7, 8]. The reduction in triglyceride, serum alanine aminotransferase (ALT) and aspartate aminotransferase (AST) levels were observed in NASH patients after 1-year dietary intervention [9]. Promrat et al. [10] also found that NASH histological activity score (NAS) was improved after 48 weeks of diet and exercise intervention. Cheng et al. [11] revealed that liver steatosis was reduced in NAFLD patients after exercise training combined with low carbohydrate diet. All above findings demonstrated that lifestyle intervention is an effective way to treat NAFLD, while the underlying epigenetic mechanism remains obscure.

Recently, epigenetic phenomena such as DNA methylation is considered to be involved in the occurrence and development of NAFLD and NASH [12–15], providing a pathway in translating environmental factors into phenotypic traits [16]. Only a few studies have reported the DNA methylation profiles after lifestyle interventions in NAFLD [17–19]. Yaskolka et al. [19] suggested that differential DNA methylation of alpha-2-macroglobulin pseudogene 1 (*A2MP1*) and calcium release activated channel regulator 2 (*ACRACR2A*) in blood could be the epigenetic markers for intrahepatic fat in NAFLD patients after diet and physical activity intervention, respectively. Yoon et al. [18] reported that DNA methylation of caspase 1 (*CASP1*) and *NADH* dehydrogenase 1 beta subcomplex 9 (*NDUFB9*) were downregulated and upregulated in liver of obese mice respectively, in response to high fat diet (HFD) compared with the control diet. Notably, DNA methylation occurs under a highly tissue specific manner [20]. In view of the difficulty of acquisition of human tissues, e.g., liver and adipose tissue, blood usually was used as a substitute to investigate whether the DNA methylation marker could reflect the signature cross tissues. And Dick et al. demonstrated that body mass index (BMI) was positively associated with DNA methylation of hypoxia inducible factor 3 alpha subunit (*HIF3A*) both in blood and adipose tissue. Nevertheless, it is still unclear what are the detailed DNA methylation mechanisms behind the long-lasting effects of separate aerobic exercise, low carbohydrate diet (LCD) or low fat diet (LFD) and the combination of exercise and diet, especially in blood, liver and adipose tissue of NAFLD and NASH.

Given the increasing evidences of the DNA methylation involvement in metabolic diseases, and considering DNA methylation playing intriguing roles in exercise and diet related prevention and treatment of NAFLD and NASH, we aim to: (1) explore genome wide DNA methylation changes before and after exercise and LCD intervention in human NAFLD; (2) investigate the effect of respective and concurrent exercise and LFD intervention on DNA methylation of candidate genes in blood of NAFLD and evaluate these effect in liver and adipose tissue of NASH mice; (3) reveal the correlations between DNA methylation of candidate gene and clinical phenotypes after intervention.

## Materials and methods

### Human trial

#### Study participants

This work is an extension of our previous study on exercise and diet intervention in NAFLD population [11, 21]. Male or female aged 50–65 years with hepatic fat content (HFC) > 5% [22], BMI ≤ 38 kg/m^2^, no alcohol consumption, no chronic cardiovascular or musculoskeletal diseases, no type I/II diabetes and no mental illness were included in this trial. And they were randomly assigned (1:1:1:1) to exercise (Ex, n = 29), LCD (LCD, n = 28) and exercise plus diet intervention (ELCD = 29), and no intervention (No = 29) groups for minimal 6 months. A subgroup comprising 32 female aged 50–65 years with NAFLD was finally included, and they were selected on the basis of HFC decline in the Ex (n = 8), LCD (n = 8) and ELCD (n = 9) groups, or HFC gain in No (n = 7) group for minimal 6 months (Additional file 1).

The Ex and ELCD groups participated an aerobic exercise training program under the supervision of exercise trainers [11]. The intensity was ranged from 60 to 75% of the maximum oxygen uptake (VO_2max_), 30 to 60 min per session which included the warm-up and cool-down exercise for 5 min, respectively, and the frequency was 2 to 3 times per week.

The LFD and ELFD groups were offered a daily lunch based on each subject’s dietary intakes and body weight, and were advised to follow an individual nutritional consultation program for breakfast and dinner [11]. The daily lunch was accounted 30–40% of their total daily energy intake, in particular, 37–40% carbohydrate, 35–37% fat and 25–27% protein.

The no intervention group (No) was suggested to keep their physical activity and dietary habits the same during the intervention.

#### Measurement

Collection of phonotypes including lifestyle and medical history information, anthropometry, body composition, HFC, abdominal subcutaneous adipose tissue (SAT) and visceral adipose tissue (VAT) were reported in our previous study [11]. Lifestyle and medical history were collected by questionnaires. Fat mass (FM) and lean mass (LM) of the whole body, and android and gynoid regions were assessed by dual-energy X-ray absorptiometry (DXA Prodigy, GE Lunar Corp., Madison, WI USA). HFC, SAT and VAT was measured by ^1^H MRS [23, 24]. BMI was calculated as weight (kg) divided by height squared (m^2^).

Insulin and glucose were assessed from glucose tolerance test (performed after overnight fasting and 2 h after the intake of 75 g glucose). Total cholesterol and triglycerides were measured from serum by conventional methods [21].

#### Genome-wide DNA methylation analysis

DNA was extracted from peripheral blood; methylation of genomic DNA was quantified using Infinium Methylation EPIC BeadChip assay (Illumina, CA, USA), which covers 865,918 CpGs [25]. After bisulfite-converting the genomic DNA samples with Zymo EZ DNA Methylation-Gold Kit (Zymo Research, Orange, CA, USA), pre- and post-amplification automated laboratory protocols were performed according to the Infinium HD methylation Assay Guide [26–28]. The BeadChips’ images were captured with the Illumina iScan. Raw methylation score for each CpG site expressed as β-values was calculated (β = intensity of the Methylated allele (M)/intensity of the Unmethylated allele (U) + intensity of the Methylated allele (M) + 100) with Genome Studio software (version 2011.1). The output Idat files were processed using ChAMP data package (version 2.8.1). After loading Idat files, several filtering steps were preformed: (1) Discard the sample with probes’ ratio above threshold (default = 0.1); (2) Filter out probes with < 3 beads in at least 5% of samples per probe and all non-CpG probes contained in this dataset; (3) Exclude all SNP-related probes, multi-hit probes or probes located in chromosome X and Y to improve the hybridization efficiency and explore the true signals [29, 30]. Next, normalization with BMIQ function was conducted to adjust the effects of type-II probe bias. To account for multiple testing and reduce the number of false positives, we applied the false discovery rate (FDR) on our genome-wide analysis of DNA methylation (q < 0.05), and CpG sites which are located in promoters (transcription start site 200 (TSS200), TSS1500 and 5 prime untranslated region (5’UTR)) were used for subsequent analysis (|Δβ|≥ 0.1), as there is incontrovertible evidence that methylation at promoters silenced gene transcription [31], thus making us focus on tissue specific DNA methylation in NAFLD and NASH after lifestyle intervention, not be distracted by the relationship between DNA methylation of different gene regions and gene transcription activity.

#### ***3D genome*** [32]

3D genome information from human embryonic stem cell [33] was built through high-throughput chromosome conformation capture (Hi-C) technology. As genes with adjacent position of chromosome had similar function [34], k-means clustering method was firstly used to cluster differentially methylated genes from the aspect of gene structure/location according to 3D genome of human cells. Then, Kyoto Encyclopedia of Genes and Genomes (KEGG) pathway enrichment analysis was performed with the R package clusterProfiler [35] and q value less than 0.05 was considered statistically significant. We also constructed the KEGG pathway gene interaction network using the STRING database, and visualize the pathways with igraph and ggraph R packages. By this double-fisted approach combined with 3D genome information and KEGG enrichment analysis, the most differentially methylated genes could be selected.

### Animal experiment

#### Experimental design

Seventy C57BL/6 male mice with specific pathogen free (SPF) grades, aged 9 weeks were used in this study. After acclimating to their environment for 1 week, weight ≥ 25 g, all animals had free access to food and tap water at a pathogen free animal care facility. At 10 weeks of age, the mice were randomly assigned to either a methionine choline deficient diet (MCD, #519580, Dyets, n = 55) or a methionine choline sufficient diet (MCS, #519581, Dyets, n = 15) group, respectively, for 4 weeks. After 4 weeks, 7 mice in each group were sacrificed when finishing an exercise training to evaluate their exercise adaptability and check whether they are NASH mice. Then NASH mice were randomly assigned to 6 groups for 4 weeks of intervention period: feeding with HFD (#D12492, Research Diets, n = 8), aerobic exercise plus HFD (EHFD, n = 8), LFD (#LF10C, Dyets, n = 8), aerobic exercise plus LFD (ELFD, n = 8), MCS diet (MCSM, n = 8), MCD diet (MCD, n = 8). The control group remained to MCS diet (MCSC, n = 8).

Exercise training was performed on a motor-driven rodent treadmill. For 5 days acclimation, mice in the EHFD and ELFD groups ran 10 min at a speed of 10 m/min on Monday, 13 m/min for 25 min on Tuesday, 14 m/min for 35 min on Wednesday, 15 m/min for 45 min on Thursday and Friday, then had rest for 2 days. In the end, mice ran 15 m/min for 45 min for 4 weeks, at a frequency of 5 day/week. Food intake and body weight were recorded twice and once a week for the entire period of the study, respectively.

After 4 weeks of intervention, the blood was obtained when the mice were sacrificed; the serum was stored at − 80 °C for the biochemical analysis. Liver and epididymal fat tissues were collected and weighed. Aliquots were snap frozen and stored at − 80 °C. Part of the liver tissue was fixed in 4% buffered paraformaldehyde and processed and embedded in paraffin for histological analysis. Liver sections were stained with haematoxylin and eosin (HE) for routine histology or with oil red O for detection of lipids.

#### Biochemical assay

Total cholesterol, triglyceride, serum ALT and AST levels were measured using automated clinical chemistry analyser (7020, HITACHI, Japan) in the Laboratory of Animal Centre, Shanghai University of Traditional Chinese Medicine.

#### DNA methylation analysis

Genomic DNA was isolated from mice liver and adipose tissue using a TIANamp Genomic DNA Kit (#DP304, TIANGEN, China). Bisulfite conversion of genomic DNA was performed using the EpiTect Fast Bisulfite Conversion Kit (#59824, Qiagen, Germany). The CpG island region was selected to analyze the CpG methylation level. Target-specific primer pairs to amplify bisulfite-treated genomic DNA were designed using the EpiDesigner tool (Agena Bioscience, Inc., San Diego, CA, USA). Primers were synthesized by Generay, and each reverse primer had a T7 promoter tag (5′-CAGTAATACGACTCACTATAGGGAGAAGGCT-3′) for transcription. The forward primer was tagged with a 10-mer (5′-AGGAAGAGAG-3′) to balance the melting temperature. The polymerase chain reaction (PCR)-amplified products were treated with shrimp alkaline phosphatase, and in vitro transcription and base-specific cleavage were performed simultaneously. The primers of *GRB2* associated binding protein 2 (*GAB2*) were as follows, forward: aggaagagagTAGATGGATTTGGGTAGTGTAATTG; reverse: cagtaatacgactcactatagggagaaggctACAACCTCCCCTCCAACCAA, which results 354 bp fragments. The resulting DNA fragments were identified by matrix-assisted laser desorption/ionization time-of-flight mass spectrometry, and EpiTYPER (Agena Bioscience, San Diego, CA, USA) was used for the quantification of CpG methylation level.

#### Quantitative real time PCR analysis (qRT-PCR)

Total RNA in liver and adipose were extracted using TRIzol reagent (#15596‐026, Invitrogen, USA) according to the manufacturer's protocol. 2 µg total RNA was used for cDNA synthesis using the Hifair® II 1st Strand cDNA Synthesis Kit (#HB200724, YEASON, China). Total cDNA was amplified using SYBR Premix Ex Taq (Tli RNaseH Plus) (#RR420A, TaKaBa, Japan). PCRs were performed using a LightCycler (Roche Diagnostics, USA). Samples were analyzed in triplicate. Gene expression was expressed as 2(^−ΔΔCt^) and normalized to the housekeeping gene *β-Actin*, forward: CCCAGCACAATGAAGATCAAGATCAT; reverse: ATCTGCTGGAAGGTGGACAGCG. The sequences of the primers used were as follows: *GAB2* (forward: ATAGGCAGAGGTGGGTCCAT; reverse: TTGCCACATGGGAGATCCAC).

#### Statistical analysis

Clinical data in human was presented as mean ± standard deviation or standard error. Paired t test and independent samples t test were adopted for the inter- and intra-group comparison. One-way ANOVA was used for comparison among multiple groups. Least significant difference (LSD) test [36] was used for pairwise comparisons between groups under the assumption that the variance was uniform. Non-normally distributed data was analyzed by converting log to normal distributed data, and non-parametric testing, i.e., Mann–Whitney U and Kruskal–Wallis H test, was used for data that cannot be converted into normal distributed data. Correlation between methylation of CpG units and serum traits or mRNA expression were calculated using Spearman correlation analysis. *P* < 0.05 was considered statistically significant.

## Results

### Human trial

#### Anthropometric and clinical measurement

General characteristics of the study participants are shown in Table 1. After 6-month intervention, HFC (p = 0.004, p = 0.005 and p = 0.012) and total FM (p = 0.001, p = 0.022 and p = 0.034) were significantly lower, while VO_2max_ (p = 0.041, p = 0.002 and p < 0.001) was higher in the Ex, LCD and ELCD groups. Weight (p < 0.001 and p = 0.012), BMI (p = 0.001 and p = 0.015) and android FM (p = 0.034 and p = 0.001) were lower both in Ex and ELCD group, while VAT (p = 0.004 and p = 0.029) decreased in Ex and LCD group. The Ex group had decreased level of gynoid FM, SAT, fasting and 2 h glucose, TG and cholesterol (p = 0.001, p = 0.011, p = 0.036, p = 0.019, p = 0.021 and p = 0.040). By contrast, the No group had increased BMI, HFC, total body and android FM (p = 0.045, p = 0.004, p = 0.003 and p = 0.035).

**Table 1:** Clinical characteristics of study participants (n = 32) with DNA methylation data both before (baseline) and after the intervention

Variables	Ex (n = 8)		LCD (n = 8)		ELCD (n = 9)		No (n = 7)		Within Groups (p value)			
	Baseline	Intervention	Baseline	Intervention	Baseline	Intervention	Baseline	Intervention	Ex	Diet	Ex + Diet	No
HFC (%)	23.6 (11.0)	11.0 (5.5)	17.2 (13.6)	11.0 (13.2)	19.2 (11.1)	6.7 (4.1)	10.3 (5.6)	15.4 (7.3)	0.004	0.005	0.012	0.004
Weight (kg)	68.4 (10.0)	64.8 (10.8)	65.1 (8.8)	63.7 (9.1)	65.9 (6.2)	64.2 (6.2)	65.2 (13.4)	66.2 (13.9)	< .001	0.098	0.012	0.059
BMI (kg/m^2^)	27.5 (3.0)	26.1 (3.4)	26.6 (3.8)	26.0 (4.0)	25.8 (2.2)	25.1 (2.3)	25.9 (3.7)	26.2 (3.9)	0.001	0.084	0.015	0.045
LM (kg)	37.9 (5.6)	37.3 (5.6)	38.1 (4.8)	37.9 (4.9)	38.2 (3.0)	37.5 (3.5)	39.3 (8.0)	38.6 (8.4)	0.092	0.535	0.113	0.058
FM_wb_ (kg)	28.4 (5.1)	25.5 (5.8)	24.9 (4.0)	23.8 (4.4)	25.7 (4.2)	24.7 (4.5)	24.6 (6.9)	26.2 (7.1)	0.001	0.022	0.034	0.003
FM_Android_ (kg)	2.84 (0.6)	2.56 (0.8)	2.42 (0.5)	2.24 (0.4)	2.43 (0.5)	2.25 (0.4)	2.41 (0.8)	2.59 (0.7)	0.034	0.059	0.001	0.035
FM_Gynoid_ (kg)	3.65 (0.8)	3.47 (0.7)	3.32 (0.9)	3.18 (0.8)	3.48 (0.8)	3.35 (0.9)	3.27 (0.8)	3.46 (0.8)	0.001	0.070	0.186	0.105
VAT (kg)	1.83 (0.6)	1.45 (0.6)	1.44 (0.6)	1.20 (0.6)	1.47 (0.7)	1.31 (0.6)	1.62 (0.8)	1.70 (0.8)	0.004	0.029	0.058	0.413
SAT (kg)	2.67 (0.8)	2.33 (0.7)	2.07 (0.5)	2.00 (0.6)	2.18 (0.7)	2.12 (0.8)	2.12 (0.6)	2.18 (0.6)	0.011	0.159	0.396	0.478
Glucose fasting (mmol/L)	5.73 (0.5)	5.26 (0.6)	5.48 (0.9)	5.58 (0.8)	5.50 (0.7)	5.48 (0.3)	5.97 (0.5)	6.11 (0.7)	0.036	0.737	0.939	0.392
Glucose 2-h (mmol/L)	9.34 (2.2)	6.63 (1.3)	8.16 (1.5)	6.54 (3.0)	8.21 (1.3)	6.98 (2.1)	8.13 (1.1)	8.60 (2.7)	0.019	0.245	0.076	0.637
Insulin fasting (µIU/mL)	25.9 (15.4)	18.4 (10.6)	14.3 (11.5)	13.9 (8.3)	20.2 (8.1)	15.9 (4.5)	16.4 (10.8)	16.4 (12.0)	0.154	0.843	0.067	0.997
HBA1c (%)	6.19 (0.3)	6.07 (0.3)	6.07 (0.3)	6.13 (0.2)	5.83 (0.5)	5.7 (0.5)	6.52 (0.2)	6.45 (0.4)	0.280	0.673	0.354	0.666
Triglyceride (mmol/L)	2.05 (1.8)	1.40 (1.2)	1.76 (0.8)	1.51 (0.5)	2.62 (1.3)	2.14 (1.1)	2.01 (1.0)	2.24 (1.3)	0.021	0.332	0.195	0.555
Cholesterol (mmol/L)	6.00 (0.7)	5.19 (0.9)	5.12 (0.9)	4.85 (0.6)	6.19 (1.2)	5.18 (0.6)	6.06 (0.9)	6.22 (0.7)	0.040	0.456	0.071	0.752
ALT (U/L)	24.0 (11.7)	16.3 (5.6)	22.6 (16.0)	18.6 (10.3)	24.6 (11.7)	22.6 (9.9)	18.7 (8.5)	30.7 (15.3)	0.232	0.533	0.511	0.116
AST (U/L)	27.5 (4.2)	19.5 (7.1)	24.4 (12.4)	23.5 (9.8)	24.7 (6.7)	23.0 (7.5)	24.6 (7.2)	28.4 (7.0)	0.081	0.887	0.663	0.496
VO_2max_ (mL/min/kg)	13.7 (4.9)	18.0 (3.9)	16.6 (3.9)	19.8 (3.1)	15.9 (4.5)	25.0 (2.9)	17.9 (4.2)	21.1 (5.3)	0.041	0.002	< 0.001	0.151

Variables are presented as mean (SD). P values are based on a paired t test

ALT: Alanine aminotransferase; AST: Aspartate aminotransferase; BMI: Body mass index; ELCD: Exercise plus low carbohydrate diet intervention; Ex: Exercise intervention; FMandroid: Android fat mass; FMgynoid: Gynoid fat mass; FMwb: Total body fat mass; HBA1c: Glycated hemoglobin; HFC: Hepatic fat content; LCD: Low carbohydrate diet; LM: Total body lean mass; No: No intervention; SAT: Abdominal subcutaneous fat content; VAT: Abdominal visceral fat content; VO_2max_: Maximal oxygen uptake

#### Differential methylation of global and individual CpG loci

**Fig. 1:**
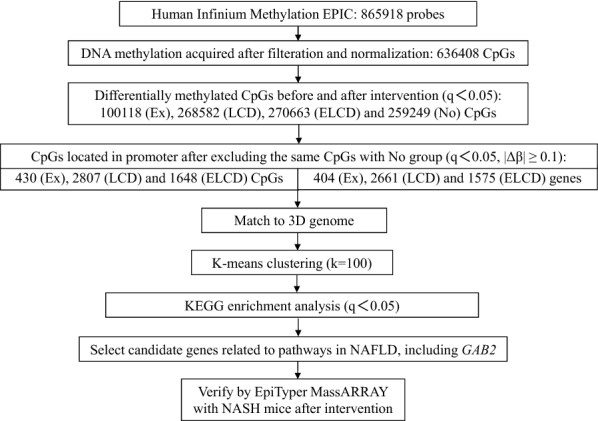
Analysis flowchart of exercise and diet intervention on DNA methylation of NAFLD in human. Ex: exercise intervention; ELCD: exercise plus low carbohydrate diet; KEGG: Kyoto encyclopedia of genes and genomes; *GAB2*: *GRB2* associated binding protein 2; LCD: low carbohydrate diet; NASH: nonalcoholic steatohepatitis; No: no intervention group

We firstly examined DNA methylation difference among the 865,918 analyzed CpGs in peripheral blood in response to Ex, LCD and ELCD intervention (Fig. 1) and estimating overlap in control group.

**Fig. 2:**
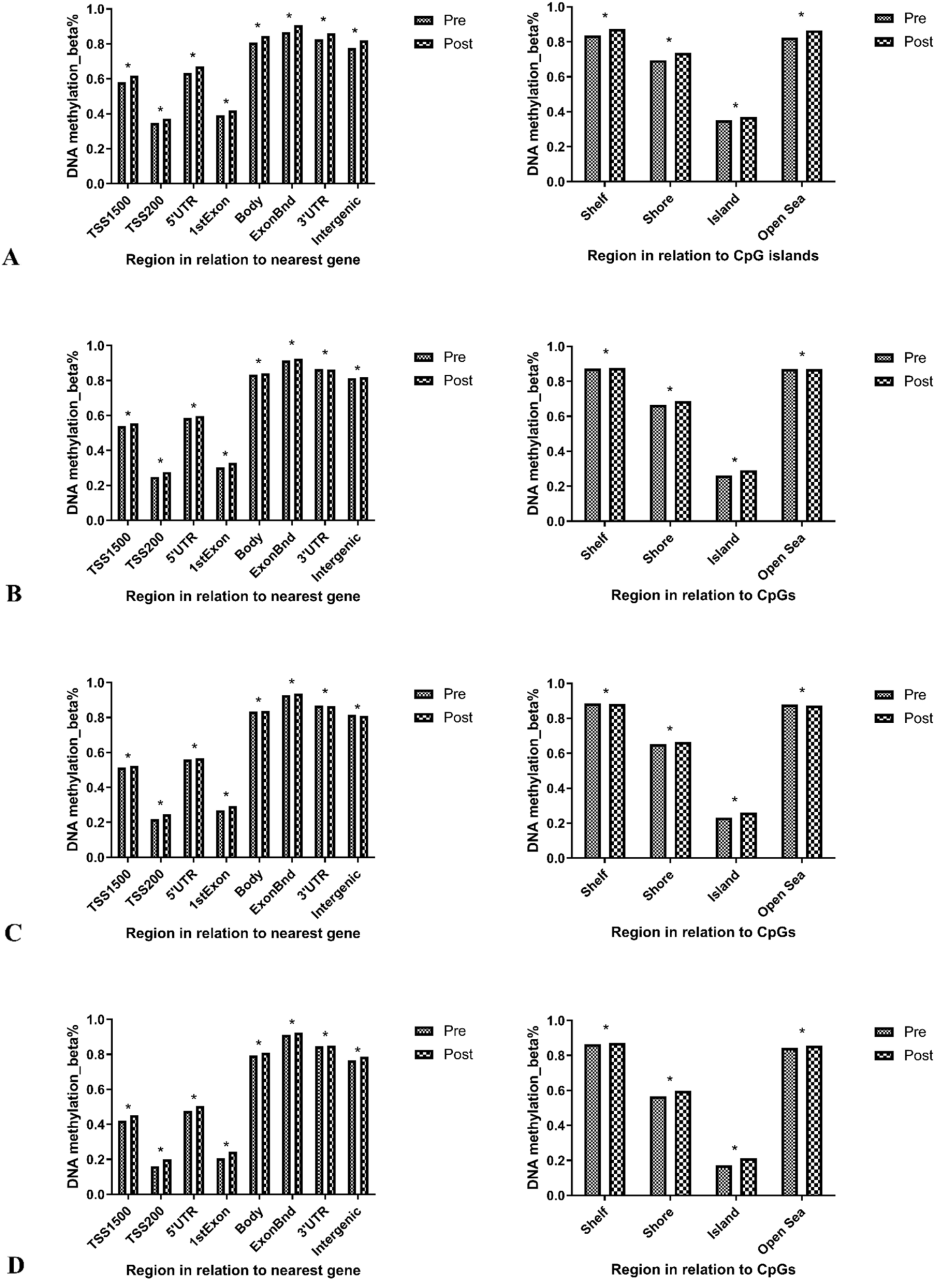
Location of analyzed CpG sites and global DNA methylation in NAFLD after intervention. **A**, **B**, **C** and **D** indicate Ex, LCD, ELCD and No groups, respectively. Pre and Post indicate samples are taken before and after intervention, respectively. Ex: exercise intervention; ELCD: exercise plus low carbohydrate diet; LCD: low carbohydrate diet; No: no intervention. *P < 0.05

After comparing the methylation of individual CpGs between baseline and follow-up, 100118, 268582, 270663 and 259249 differentially methylated CpGs (q < 0.05) were identified in Ex, LCD and ELCD and No groups, respectively. A pattern of methylation across the genome with the least methylation in promoter regions and the most methylation in the ExonBnd and 3 prime untranslated region (3’UTR) was observed. Significantly lower methylation levels were observed in pre-intervention than post-intervention for all gene regions except 3’UTR in LCD and ELCD groups (p < 0.05). Furthermore, the least methylation in CpG island and most methylation in the shelves and open sea were found, which was significantly more methylated at post-intervention than pre-intervention except for the open sea region in LCD and ELCD groups (p < 0.05) (Fig. 2).

**Fig. 3:**
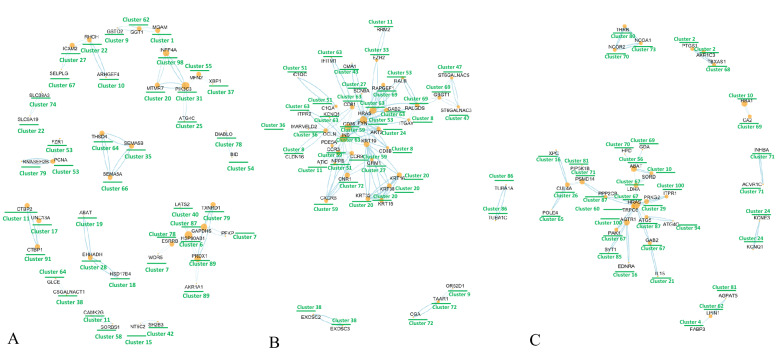
Network of differentially methylated genes in NAFLD after Ex, LCD and ELCD intervention. **A**, **B** and **C** indicate Ex, LCD and ELCD groups, respectively. Ex: exercise intervention; ELCD: exercise plus low carbohydrate diet; LCD: low carbohydrate diet

#### 3D genome territorial and k-means clustering

We further selected 430, 2807 and 1648 differentially methylated CpGs within promoters, corresponding to 404, 2661 and 1575 (q < 0.01, |Δβ|≥ 0.1) genes in Ex, LCD and ELCD group, respectively, after excluding the overlap CpGs with No group. Then, the above differentially methylated genes were assigned 3D genome coordinates according to the chromosome number and TSS using our 3D modeling methods [33]. These differentially methylated genes with 3D genome information were separated into 100 clusters by K-means clustering algorithm (optimal cluster numbers were pre-investigated).

#### Differential methylation at the pathway level

To better understand the biological function of the differentially methylated genes, KEGG enrichment analysis was conducted in three intervention groups (Additional file 2) and the relevant pathways, i.e., lipid metabolism (e.g., fat digestion and absorption, fatty acid degradation), carbohydrate metabolism (e.g., glycolysis, fructose and mannose metabolism), inflammation (e.g., *IL-17* signaling pathway and *TNF* signaling pathway) and key signaling pathways (e.g., *PPAR* signaling pathway and *TGF-beta* signaling pathway) were identified.

To visualize the gene–gene interaction, graphs with clusters being marked according to 3D genome coordinates were displayed (Fig. 3). And we observed that genes, e.g., *GAB2* and *HRas* proto-oncogene (*HRAS*), insulin (*INS*) and *CD81*, which were included in the pathways related to lipid metabolism and inflammation were belonged to cluster 63 after LCD intervention. Genes including *GAB2*, *HRAS*, *P21* activated kinase 1 (*PAK1*) and lactate dehydrogenase A (*LDHA*), that are related to lipid and carbohydrate metabolism, were assigned to cluster 67 after ELCD intervention. Notably, both *GAB2* were hypomethylated and *HRAS* was hypermethylated in response to LCD and ELCD intervention (Table 2).

**Table 2:** Specific information on CpGs of promoters in *GAB2* and *HRAS* in NAFLD patients after diet and exercise plus diet intervention

		Pre_average methylation	Post_average methylation	Δβ	p value	q value	Chromosome	Feature	CGI
LCD	*GAB2*								
	cg02802179	0.982	0.934	− 0.048	0.007	0.020	11	TSS1500	Opensea
	cg04116507	0.880	0.928	0.048	0.009	0.025	11	5’UTR	Opensea
	cg13087871	0.630	0.514	− 0.117	0.012	0.031	11	5’UTR	Opensea
	*HRAS*								
	cg05798318	0.139	0.242	0.103	0.005	0.016	11	TSS1500	Island
	cg02606081	0.100	0.146	0.046	0.011	0.029	11	TSS1500	Island
	cg05508592	0.054	0.070	0.016	0.018	0.043	11	5'UTR	Island
	cg06920740	0.076	0.064	− 0.012	0.019	0.045	11	5'UTR	Island
ELCD	*GAB2*								
	cg22494377	0.934	0.876	− 0.058	0.005	0.016	11	5’UTR	Opensea
	cg13087871	0.657	0.528	− 0.129	0.011	0.030	11	5’UTR	Opensea
	*HRAS*								
	cg05798318	0.244	0.372	0.128	0.014	0.036	11	TSS1500	Island

CGI: cytosine-guanine dinucleotide island; CpGs: cytosine-guanine dinucleotides; *GAB2*: *GRB2* associated binding protein 2; NAFLD: nonalcoholic fatty liver disease; *HRAS*: *HRas* proto-oncogene; ELCD: exercise plus low carbohydrate diet; LCD: low carbohydrate diet; TSS1500: transcription start site 1500 base pair; 5’UTR: 5 prime untranslated regions

From the aspect of gene structure and gene function by 3D genome information and KEGG analysis respectively, we observed that *GAB2* and *HRAS* were assigned to the same cluster after LCD and ELCD intervention respectively (Fig. 3), and involved in phospholipase D signaling pathway (Additional file 2), which are highly related to lipid metabolism [37]. Combine this finding with *GAB2* being regarded as a candidate gene regulating hepatic steatosis and steatohepatitis [38, 39], we decided to firstly evaluate if *GAB2* belongs to genes that showing decreased DNA methylation in the promoter region coupled with increased mRNA expression in liver and adipose of NASH after LFD or ELFD intervention; animal experiment followed by human trial was thus carried on.

### Animal experiment

#### Basic traits in NASH mice before and after intervention

After 4 weeks of MCD diet feeding, the histology of liver was examined to check whether NASH model was successful by HE staining (Additional file 3). Then NASH mice were assigned to intervene for 4 weeks. And liver histological characteristics were stained with HE (Additional file 4) to check whether lifestyle intervention decreases the triglyceride accumulation and improves the inflammation symptom in NASH. Furthermore, oil red O staining was also used to detect the lipid change in HFD, EHFD, LFD, ELFD and MCD groups (Additional file 5). And the reduced vesicular degeneration was seen after LFD and ELFD intervention with both HE and oil red O staining.

Basic traits were shown in the additional files (Additional files 6, 7, 8). Weight of all the intervention groups i.e., HFD, EH, LFD and ELFD were much higher than MCD group. There was a significant increase in ALT and AST level, but decrease in total cholesterol and triglyceride of MCD group compared with intervention groups. The cholesterol level in HFD was much higher than EHFD, but triglyceride was significantly lower in HFD than EHFD (p < 0.05). No significant differences in cholesterol and triglyceride were found in LFD and ELFD groups.

To check diet effect and exercise plus diet effect on DNA methylation of *GAB2* in NASH, DNA methylation profiling by EpiTyper MassARRAY and relative mRNA expression profiling by qRT-PCR were conducted for MCD, HFD, LFD and ELFD groups.

#### Diet effect on DNA methylation and mRNA expression of GAB2 in liver and adipose of NASH mice

**Fig. 4:**
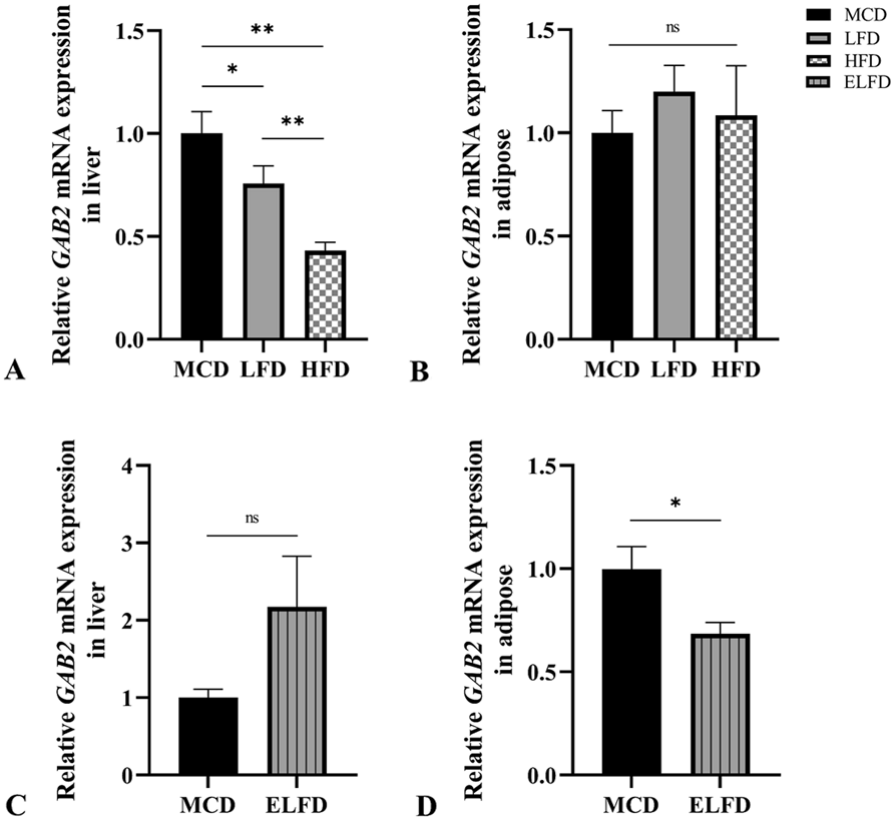
Relative mRNA expression level of GAB2 in mice liver and adipose tissue. A and B indicate relative mRNA expression levels of GAB2 among MCD, LFD and HFD groups in liver and adipose, respectively. P values in **A** and **B** are based on one way ANOVA. **C** and **D** indicate relative mRNA expression levels of GAB2 between MCD and ELFD groups in liver and adipose, respectively. P values in C and D are based on independent samples T test. *P < 0.05; **P < 0.01; ns: no significance

In liver, methylation level of CpG_1.2 within *GAB2* were much lower in MCD than LFD and HFD groups (p = 0.043) (Table 3). The relative mRNA expression of *GAB2* in MCD group was higher than LFD and HFD (p < 0.05) (Fig. 4).

**Table 3:** *GAB2* methylation values (%) for each CpG unit among four groups in liver and adipose tissues of mice

CpGs	Liver (Methylation%)						Adipose (Methylation%)					
	MCD	LFD	HFD	ELFD	P1	P2	MCD	LFD	HFD	ELFD	P3	P4
CpG_1.2	0.00	3.80	4.00	29.50	0.043	0.114	1.71	1.00	1.83	1.86	0.327	0.620
CpG_3	0.00	0.40	5.33	1.00	0.075	0.686	2.29	3.67	0.33	2.57	0.288	1.000
CpG_5	8.00	4.20	10.17	0.00	0.459	0.114	0.43	1.89	5.83	1.00	0.754	0.318
CpG_6.7	9.25	14.00	15.00	4.00	0.522	0.400	8.71	18.33	5.67	2.57	0.044	0.001
CpG_8	2.25	3.25	5.67	31.50	0.812	0.200	1.14	1.88	4.33	1.43	0.360	1.000
CpG_9	6.25	8.00	0.83	6.00	0.054	0.686	1.14	4.11	2.17	5.00	0.216	0.073
CpG_10.11.12	3.75	9.00	6.17	8.00	0.498	0.629	3.14	5.33	3.00	8.43	0.184	0.002
CpG_13.14.15	2.50	3.80	2.67	18.25	0.400	0.114	2.29	3.11	2.33	6.29	0.842	0.001
CpG_16.17	3.00	3.00	3.00	20.75	0.608	0.057	3.43	3.44	2.33	6.86	0.303	0.053
CpG_18.19	2.00	2.60	3.50	15.75	0.427	0.029	2.00	1.44	2.17	6.57	0.203	0.001
CpG_20.21	3.75	2.60	9.83	10.00	0.342	0.200	0.71	3.67	7.67	1.86	0.598	0.073
CpG_23.24	3.00	4.00	3.33	6.25	0.788	0.343	3.29	3.78	2.83	4.57	0.466	0.902
CpG_25.26.27.28	7.25	10.40	10.17	13.00	0.134	0.200	8.86	10.33	10.00	11.29	0.359	0.017
CpG_30	2.25	3.00	3.50	6.75	0.918	0.343	2.57	2.67	1.83	9.86	0.155	0.001
CpG_31	5.25	1.60	5.17	3.50	0.308	0.486	2.71	4.78	2.86	10.29	0.670	0.001
CpG_33	0.75	1.20	2.33	0.50	0.386	0.486	1.43	1.56	0.83	3.57	0.225	0.026
Average CpG	3.70	4.68	5.69	12.13	0.513	0.057	2.87	4.59	3.03	5.25	0.048	0.001

P1 and P3 indicate p value among MCD, LFD and HFD in liver and adipose, respectively. P2 and P4 indicate p value between MCD and ELFD in liver and adipose, respectively

CpGs: cytosine-guanine dinucleotides; *GAB2*: *GRB2* associated binding protein 2; ELFD: exercise plus low fat diet; HFD: high fat diet; LFD: low fat diet; MCD: methionine choline deficient

In adipose, CpG_6.7 and average methylation level of *GAB2* were much higher in LFD group than MCD and HFD groups (p = 0.048) (Table 3). While there was no significant difference in relative mRNA expression of *GAB2* among the three groups (Fig. 4).

#### Exercise plus diet effect on DNA methylation and mRNA expression of GAB2 in liver and adipose of NASH mice

In liver, a significant increase in methylation level of CpG_18.19 within *GAB2* of ELFD group was found when comparing with MCD group (p = 0.029) (Table 3). But there was no significant difference in relative mRNA level of *GAB2* between these two groups (Fig. 4).

In adipose, average methylation level and seven specific CpG units including CpG_10.11.12, CpG_13.14.15, CpG_18.19, CpG_25.26.27.28, CpG_30, CpG_31, CpG_33 of *GAB2* showed significantly increase in ELFD group than MCD group (p < 0.05), except CpG_6.7 (Table 3). And the expression of *GAB2* in EL group was significantly lower than MCD group (p = 0.045) (Fig. 4).

#### Correlation between methylation of CpG units and serum traits in NASH mice after intervention

In liver, methylation level of CpG_18.19 was positively correlated with ALT and AST, but negatively correlated with cholesterol and triglyceride; methylation level of CpG_5 was positively correlated with ALT and AST, but negatively correlated with cholesterol; methylation level of CpG_1.2 and CpG_25.26.27.28 were both positively correlated with cholesterol (p < 0.05) (Table 4).

**Table 4:** Spearman correlation coefficients of methylation levels of *GAB2* and metabolic traits in mice

CpG units	Liver				Adipose			
	ALT	AST	Cholesterol	Triglyceride	ALT	AST	Cholesterol	Triglyceride
CpG_1.2	− 0.518	− 0.573	0.741*	0.478	− 0.091	− 0.148	− 0.154	− 0.309
CpG_3	− 0.247	− 0.577	0.083	0.255	0.177	− 0.109	− 0.156	0.086
CpG_5	0.788*	0.788*	− 0.895*	− 0.723	− 0.406	− 0.535*	0.043	0.451
CpG_6.7	0.252	0.36	− 0.318	− 0.359	0.558	0.653*	− 0.765**	− 0.645*
CpG_8	− 0.319	− 0.396	0.475	0.263	0.007	− 0.025	0.144	− 0.224
CpG_9	− 0.196	0.123	0.198	− 0.108	− 0.644*	− 0.656*	0.247	0.402
CpG_10.11.12	− 0.487	− 0.126	0.518	0.349	− 0.706**	− 0.695**	0.727**	0.681**
CpG_13.14.15	− 0.524	− 0.319	0.681	0.481	− 0.839**	− 0.830**	0.684**	0.667**
CpG_16.17	− 0.515	− 0.371	0.663	0.364	− 0.591*	− 0.629*	0.177	0.427
CpG_18.19	− 0.743*	− 0.790*	0.819*	0.920**	− 0.687**	− 0.691**	0.758**	0.552*
CpG_20.21	− 0.349	− 0.446	0.085	0.056	− 0.420	− 0.605*	0.328	0.498
CpG_23.24	− 0.476	− 0.381	0.575	0.160	− 0.066	− 0.213	− 0.389	0.183
CpG_25.26.27.28	− 0.458	− 0.386	0.752*	0.342	− 0.717**	− 0.693**	0.386	0.378
CpG_30	− 0.615	− 0.615	0.261	0.342	− 0.721**	− 0.727**	0.683**	0.463
CpG_31	0.024	0.171	− 0.160	− 0.340	− 0.773**	− 0.835**	0.609*	0.620*
CpG_33	0.091	0.378	− 0.190	− 0.686	− 0.695**	− 0.628*	0.424	0.378
AverageCpG	− 0.571	− 0.619	0.623	0.516	− 0.805**	− 0.832**	0.573*	0.491

ALT: alanine aminotransferase; AST: aspartate aminotransferase; CpG: cytosine-guanine dinucleotide; *GAB2*: *GRB2* associated binding protein

*P < 0.05; **P < 0.01

In adipose, negative relationship between average methylation of CpG units and specific methylation of CpG units (i.e., CpG_9, CpG_10.11.12, CpG_13.14.15, CpG_16.17, CpG_18.19, CpG_25.26.27.28, CpG_30, CpG_31 and CpG_33) and ALT and AST were observed; average methylation level of CpG and specific CpG units (i.e., CpG_10.11.12, CpG_13.14.15, CpG_18.19, CpG_20.21, CpG_30 and CpG_31) was positively related to cholesterol, of these, CpG_10.11.12, CpG_13.14.15, CpG_18.19 and CpG_31 were also positively correlated with triglyceride. Methylation of CpG_10.11.12 was negatively correlated with mRNA expression of *GAB2* (p < 0.05) (Table 4).

## Discussion

This study highlighted the respective and combined effects of exercise and diet intervention on the blood DNA methylation in NAFLD, and evaluated whether these interventions protected liver and adipose against NASH via DNA methylation. Our results revealed that (1) lifestyle intervention can trigger genome-wide differential DNA methylation changes in human NAFLD; (2) both LCD and ELCD intervention on human NAFLD can induce same DNA methylation alterations at critical genes in blood, e.g., *GAB2*, which was also validated in liver and adipose of NASH mice after LFD and ELFD intervention; (3) methylation of CpG units (i.e., CpG_10.11.12) inversely correlated with mRNA expression *GAB2* in adipose tissue of NASH mice after ELFD intervention.

The NAFLD is a disorder that extends from simple hepatic steatosis to NASH, which can be modeled by feeding MCD diet in mice to induce the elevation of aminotransferase and concurrent changes of hepatic histology with steatosis, hepatocyte ballooning and inflammation, and fibrosis [40, 41], and these histological changes are similar with those seen in human NASH. Notably, this complex disease cannot be solely explained by genetic and epigenetic factors. DNA methylation, which affects gene expression without changing the nucleotide sequence, acts as underling mechanistic modification to impact on the disease [42]. Moreover, DNA methylation can be affected by environmental factors such as exercise and diet, and differs significantly among skeletal muscle, adipose and liver tissues [43, 44]. Considering the applicability and effectiveness, LCD (40% fat and 40% carbohydrate) was chosen to improve NAFLD in human [45, 46], and LFD (10% fat and 70% carbohydrate) was used in NASH mice in the present study. Increased DNA methylation level of CpG island within *GAB2* in adipose of mice NASH after LFD and ELFD intervention was observed, which was in line with the result from NAFLD human after LCD and ELCD intervention, as a decrease in DNA methylation of the promoter within *GAB2* has shown. These findings strongly suggest that DNA methylation modification plays a critical role in human NAFLD and mice NASH fed a MCD diet responding to the lifestyle intervention.

To explore the optimal lifestyle intervention for NAFLD and NASH via influence on DNA methylation, respective and concurrent exercise and diet intervention were both applied in this study. The results revealed the mRNA expression of *GAB2* was repressed corresponding to the upregulated DNA methylation in NASH after ELFD intervention. Therefore, ELFD intervention was the preferred effective strategy for the treatment of NASH in this study. This discovery is in line with the report of Eckard et al. [47] that the combined intervention with exercise and diet improved liver histology, rather than the sole exercise intervention, and moreover, Goodpaster et al. [48] found that there was an addition of exercise on the reduction of HFC and waist circumstance in the obese. In contrast, Gepner et al. [49] showed that no additive effects of moderate physical activity upon Mediterranean diet and LFD intervention on HFC of NAFLD; Andersson et al. [50] reported that effects on HFC was independent of exercise or exercise plus diet interaction. More research is needed to explain these inconsistencies and elucidate the role of exercise or exercise and diet intervention in NAFLD, especially focusing on the DNA methylation mechanism behind these conditions, as this epigenetic modification is dynamically regulated by environmental factors and subsequently affect diseases.

Considering that CpG sites within the promoter region of human *GAB2* gene were reported to be hypomethylated in the blood of NAFLD patients in response to LCD and ELCD, and *GAB2* deletion prevented hepatic steatosis induced by HFD and steatohepatitis induced by MCD diet in mice [38], in this study, *GAB2* in the LFD vs. MCD or ELFD vs. MCD comparison was selected to examine its DNA methylation and gene expression in liver and adipose of NASH mice after LFD or ELFD intervention. Regarding the function of *GAB2*, Chen et al. [38] showed that *GAB2* could promote lipid accumulation in HepG2 cells in vitro, and Wang et al. [39] found that increase of *GAB2* expression in adipose was induced by HFD in mice. *GAB2* plays important roles in differentiation, proliferation and cell migration in multiple cells [51] by recruiting factors, e.g., Ras, p85 and Shp2 [52, 53]. Although no significant difference in *GAB2* mRNA expression in liver between ELFD and MCD groups was found in this study, mRNA expression level in MCD group was much higher than ELFD, in accord with DNA methylation in MCD group was significantly lower than ELFD in adipose. This suggests that ELFD could modulate *GAB2* methylation and expression status of adipose in NASH.

Liver disfunction was assessed by serum measurements of liver enzymes ALT and AST. The elevation of these enzymes reflects liver damage caused by either cell death or a transient leak [54, 55]. A positive correlation between average methylation of *GAB2* in adipose and ALT or AST was demonstrated, and both ALT and AST level were much lower after LFD and ELFD intervention in this study, indicating a role of adipose tissue *GAB2* in liver damage. Nevertheless, only methylation of CpG_5 and CpG_18.19 of *GAB2* was associated with ALT and AST in liver, which might result from the tissue specific changes between liver and adipose in NASH after LFD and ELFD intervention, as NASH and these lifestyle interventions can make it difficult to not only identify the pathological or adaptive role of DNA methylation events but also to recognize the primary benefits that triggers secondary or systemic changes of these stimulation. In addition, Nano et al. [56] has shown that ALT, gamma-glutamyl transferase and triglyceride were associated with methylation of *SLC7A11* in blood, which was reported to be linked to adiposity [57]. These findings gave indirect evidence that DNA methylation could be a contributor to the development or prevention of liver diseases.

It is well known that most CpG islands are not methylated when located at TSS, and methylation of CG island at TSS is associated with silenced gene expression [31]. Most gene bodies have poor CpG sites, but can still be extensively methylated, making the role of gene body methylation in mammal very intriguing. It has been reported that there is a positive correlation between gene body methylation and active transcription in animal genomes [58], and that methylation blocks the start of transcription rather than elongation. Our results were consistent with above findings which showed that methylation of CpG island of *GAB2* was upregulated via ELFD in NASH mice, and mRNA expression of *GAB2* was downregulated. It is thus conjectured that methylation of CpG island in *GAB2* might interrupt the start of transcription activity and thus inhibit the mRNA expression of *GAB2*.

This study focuses on two main contributions. Firstly, a quantitative methylation analysis via Sequenom’s MassARRAY system was employed to validate the DNA methylation change of *GAB2*, which was screened with Infinium Methylation EPIC Bead Chip assay on a genome wide scale in this study to increase the reliability. Secondly, respective and combined exercise and diet intervention were conducted as strategies to combat NAFLD and NASH, and the importance of concurrent intervention (i.e., ELFD) which effectively affected DNA methylation and mRNA expression of *GAB2* in adipose of NASH mice was revealed. Some limitations of this study include: firstly, the human sample size is relatively small, and the result was not replicated in an independent population, and more studies with larger sample sizes are needed to confirm our findings; secondly, because of the lack of RNA sample, this study may not provide the information of expression data of *GAB2* in human; thirdly, only CpG sites of promoters in human trial were included for the screening of differentially methylated genes, and methylation in other gene regions may also be affected by the exposure of lifestyle intervention in NAFLD; lastly, DNA methylation associations with NAFLD in response to lifestyle intervention could be confounded by different cell types in blood, and adjusting for changes in cell composition in future studies is needed. However, the validation of differentially methylated gene *GAB2* in liver and adipose tissue in NASH mice may be a better substitute for replication in another population because epigenetic modifications are rather tissue specific, and furthermore verified the modification of DNA methylation of *GAB2* may also be a target for the treatment of NASH, not only NAFLD.

## Conclusions

We highlighted the susceptibility of DNA methylation in *GAB2* to ELFD intervention, through which exercise and diet can protect against the progression of NAFLD and NASH on the genome level. We also demonstrated that the DNA methylation variation in blood could mirror epigenetic signatures in target tissues of important biological function, i.e., liver and adipose tissue. We also clarified that the association between DNA methylation of CpG units in *GAB2* and ALT, AST, total cholesterol and triglyceride, which was a step towards understanding the epigenetic programming of the disease development and identifying new epigenetic molecular targets to avert potential NAFLD, NASH and relevant liver diseases consequences.

## Supplementary Information

**Additional file 1: Fig. S1.** Flow chart of exercise and diet intervention on DNA methylation of NAFLD in human. ^1^Ex, exercise intervention; ELCD, exercise plus low carbohydrate diet; LCD, low carbohydrate diet; NAFLD, nonalcoholic fatty liver disease; No, no intervention.

**Additional file 2: Table S1.** KEGG pathway analysis in NAFLD after lifestyle intervention (q < 0.05).

**Additional file 3: Fig. S2.** Histological assessment of liver between MCD and MCS groups. ^1^MCD, methionine choline deficiency diet (4 weeks); MCS, methionine choline sufficient diet (4 weeks). Scale bars = 100 μm.

**Additional file 4: Fig. S3.** Histological assessment of liver in NASH mice after intervention stained with HE. ^1^Intervention groups: EHFD, exercise plus high fat diet; ELFD, exercise plus low fat diet; HE, haematoxylin and eosin; HFD, high fat diet; LFD, low fat diet; MCSM, methionine choline sufficient diet (4 weeks). None intervention groups: MCD, methionine choline deficiency diet (8 weeks); MCSC, methionine choline sufficient diet (8 weeks). Scale bars = 100 μm.

**Additional file 5: Fig. S4.** Histological assessment of liver in NASH mice after intervention stained with oil red O. ^1^Intervention groups: EHFD, exercise plus high fat diet; ELFD, exercise plus low fat diet; HFD, high fat diet; LFD, low fat diet; intervention groups: MCD, methionine choline deficiency diet (8 weeks). Scale bars = 100 μm.

**Additional file 6: Table S2.** Clinical traits of NASH mice after intervention. ^1^Intervention groups: EHFD, exercise plus high fat diet; ELFD, exercise plus low fat diet; HFD, high fat diet; LFD, low fat diet; MCSM, methionine choline sufficient diet (4 weeks). None intervention groups: MCD, methionine choline deficiency diet (8 weeks); MCSC, methionine choline sufficient diet (8 weeks).

**Additional file 7: Fig. S5.** Weight comparison among different groups in NASH mice. ^1^Intervention groups: EHFD, exercise plus high fat diet; ELFD, exercise plus low fat diet; HFD, high fat diet; LFD, low fat diet; MCSM, methionine choline sufficient diet (4 weeks). None intervention groups: MCD, methionine choline deficiency diet (8 weeks); MCSC, methionine choline sufficient diet (8 weeks).

**Additional file 8: Fig. S6.** Clinical traits comparison among different groups in NASH mice. ^1^Intervention groups: EHFD, exercise plus high fat diet; ELFD, exercise plus low fat diet; HFD, high fat diet; LFD, low fat diet; MCSM, methionine choline sufficient diet (4 weeks). None intervention groups: MCD, methionine choline deficiency diet (8 weeks); MCSC, methionine choline sufficient diet (8 weeks).

## Data Availability

Not applicable.

## Abbreviations

ALT: Alanine aminotransferase
AST: Aspartate aminotransferase
BMI: Body mass index
Ex: Exercise
*BCAT1*: Branched-chain amino-acid transaminase 1
*CASP1*: Caspase 1
EHFD: Exercise plus high fat diet
ELFD: Exercise plus low fat diet
ELCD: Exercise plus low carbohydrate diet
FM: Fat mass
*FGFR2*: Fibroblast growth factor receptor 2
*GAB2*: *GRB2* Associated binding protein 2
HE: Haematoxylin and eosin
HFC: Hepatic fat content
HFD: High fat diet
*HRAS*: *HRas* Proto-oncogene
*INS*: Insulin
KEGG: Kyoto encyclopedia of genes and genomes
*LDH*A: Lactate dehydrogenase A
LCD: Low carbohydrate diet
LFD: Low fat diet
LM: Lean mass
VO_2max_: Maximum oxygen uptake
MCD: Methionine choline deficient
MCS: Methionine choline sufficient
*NDUFB9*: *NADH* Dehydrogenase 1 beta subcomplex 9
No: No intervention
NAFLD: Nonalcoholic fatty liver disease
NASH: Nonalcoholic steatohepatitis
*PAK1*: *P21* Activated kinase 1
*PPARa* and *PPARγ*: Peroxisome proliferator-activated receptor-alpha and -gamma
*PDGFA*: Platelet-derived growth factor subunit A
qRT-PCR: Quantitative reverse transcription polymerase chain reaction analysis
SPF: Specific pathogen free
TSS: Transcription start site
SAT: Subcutaneous adipose tissue
UTR: Untranslated region
VAT: Visceral adipose tissue
