# Supplementary material for: Effect of exercise and diet intervention in NAFLD and NASH via GAB2 methylation
Source: Cell Biosci. 2021 Nov 4;11:189. doi: 10.1186/s13578-021-00701-6 (PMC8569968; doi:10.1186/s13578-021-00701-6)
Supplement: Supplementary file 1 — Additional file 1: Fig. S1. Flow chart of exercise and diet intervention on DNA methylation of NAFLD in human. 1Ex, exercise intervention; ELCD, exercise plus low carbohydrate diet; LCD, low carbohydrate diet; NAFLD, nonalcoholic fatty liver disease; No, no intervention. [file 13578_2021_701_MOESM1_ESM.pptx]

## Slide 1
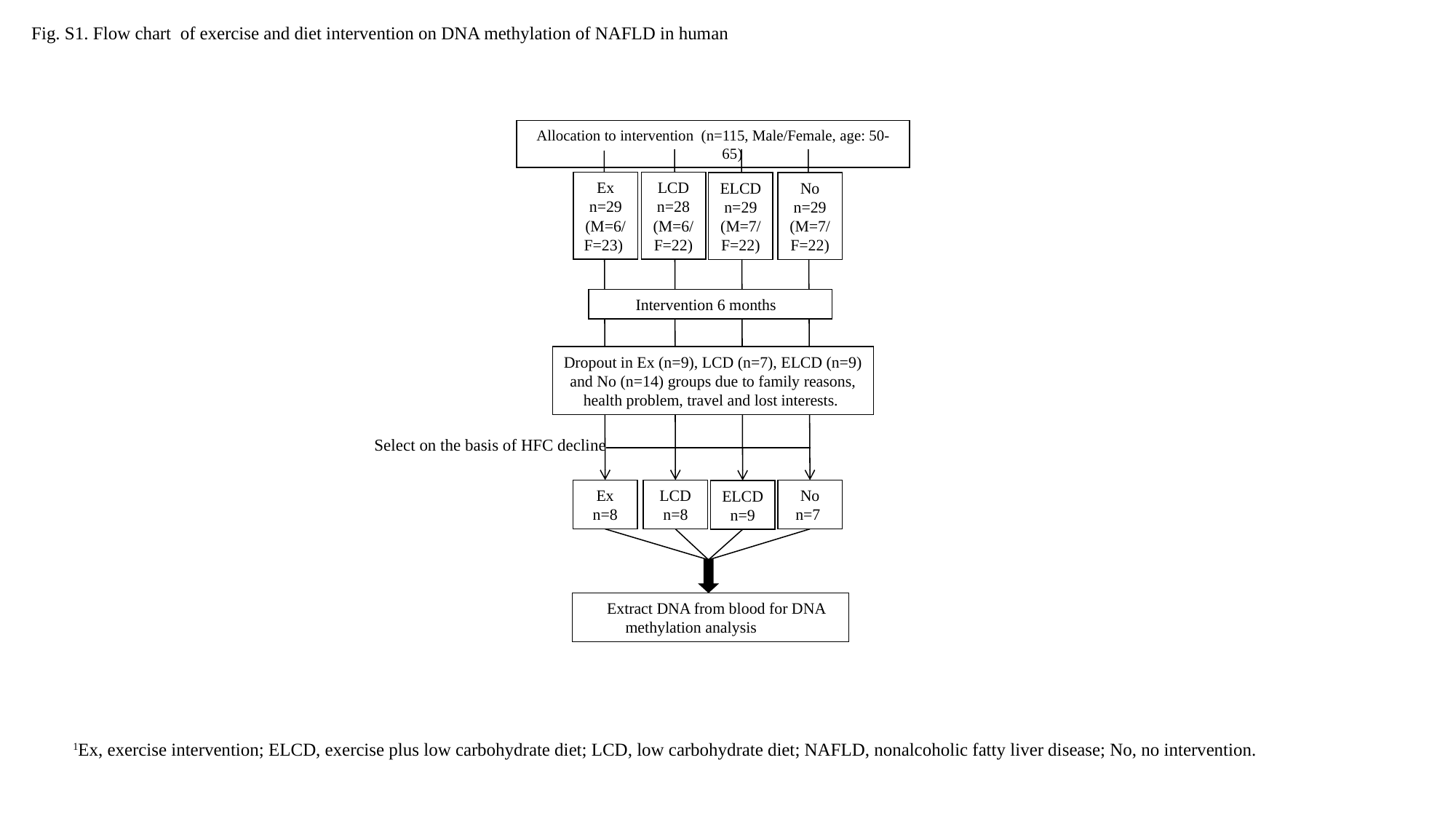

Fig. S1. Flow chart of exercise and diet intervention on DNA methylation of NAFLD in human
Allocation to intervention (n=115, Male/Female, age: 50-65)
Ex
n=29 (M=6/
F=23)
LCD
n=28 (M=6/
F=22)
No
n=29 (M=7/
F=22)
ELCD
n=29 (M=7/F=22)
 Intervention 6 months
Dropout in Ex (n=9), LCD (n=7), ELCD (n=9) and No (n=14) groups due to family reasons, health problem, travel and lost interests.
Select on the basis of HFC decline
Ex
n=8
LCD
n=8
No
n=7
ELCD
n=9
 Extract DNA from blood for DNA methylation analysis
1Ex, exercise intervention; ELCD, exercise plus low carbohydrate diet; LCD, low carbohydrate diet; NAFLD, nonalcoholic fatty liver disease; No, no intervention.
