# Supplementary material for: Effect of exercise and diet intervention in NAFLD and NASH via GAB2 methylation
Source: Cell Biosci. 2021 Nov 4;11:189. doi: 10.1186/s13578-021-00701-6 (PMC8569968; doi:10.1186/s13578-021-00701-6)
Supplement: Supplementary file 3 — Additional file 3: Fig. S2. Histological assessment of liver between MCD and MCS groups. 1MCD, methionine choline deficiency diet (4 weeks); MCS, methionine choline sufficient diet (4 weeks). Scale bars = 100 μm. [file 13578_2021_701_MOESM3_ESM.pptx]

## Slide 1
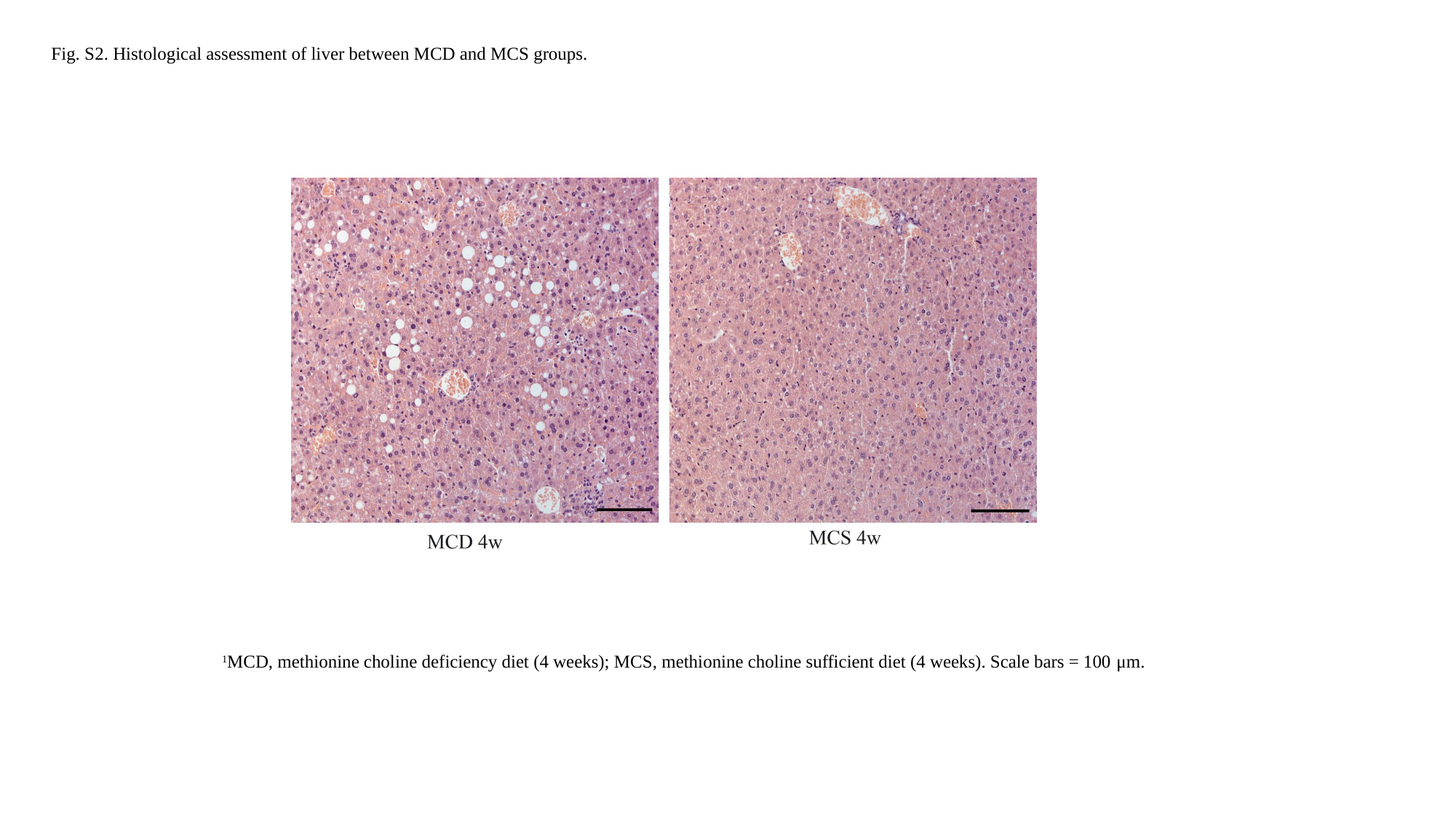

Fig. S2. Histological assessment of liver between MCD and MCS groups.
1MCD, methionine choline deficiency diet (4 weeks); MCS, methionine choline sufficient diet (4 weeks). Scale bars = 100 μm.
