# Supplementary material for: Effect of exercise and diet intervention in NAFLD and NASH via GAB2 methylation
Source: Cell Biosci. 2021 Nov 4;11:189. doi: 10.1186/s13578-021-00701-6 (PMC8569968; doi:10.1186/s13578-021-00701-6)
Supplement: Supplementary file 4 — Additional file 4: Fig. S3. Histological assessment of liver in NASH mice after intervention stained with HE. 1Intervention groups: EHFD, exercise plus high fat diet; ELFD, exercise plus low fat diet; HE, haematoxylin and eosin; HFD, high fat diet; LFD, low fat diet; MCSM, methionine choline sufficient diet (4 weeks). None intervention groups: MCD, methionine choline deficiency diet (8 weeks); MCSC, methionine choline sufficient diet (8 weeks). Scale bars = 100 μm. [file 13578_2021_701_MOESM4_ESM.pptx]

## Slide 1
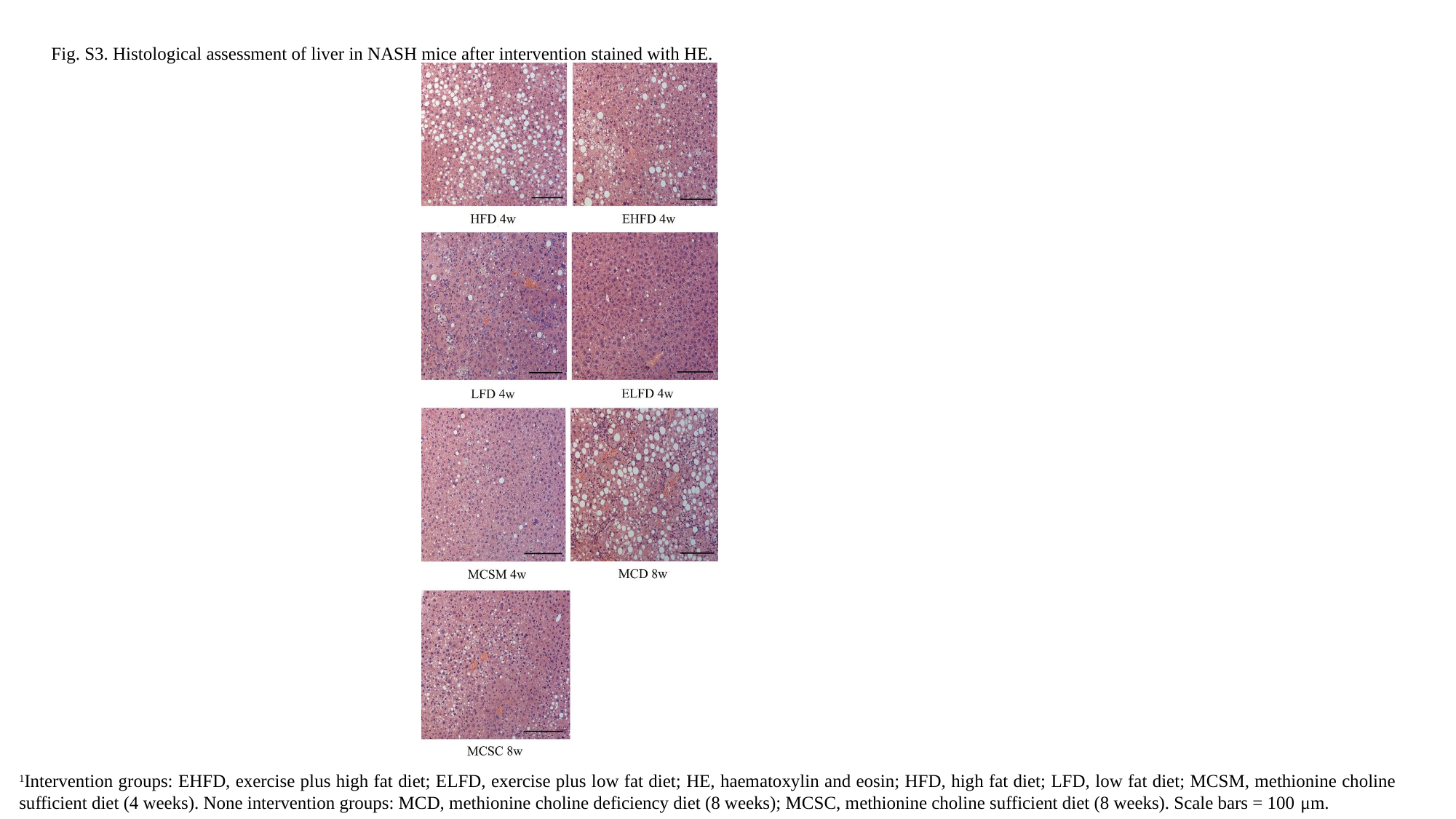

Fig. S3. Histological assessment of liver in NASH mice after intervention stained with HE.
1Intervention groups: EHFD, exercise plus high fat diet; ELFD, exercise plus low fat diet; HE, haematoxylin and eosin; HFD, high fat diet; LFD, low fat diet; MCSM, methionine choline sufficient diet (4 weeks). None intervention groups: MCD, methionine choline deficiency diet (8 weeks); MCSC, methionine choline sufficient diet (8 weeks). Scale bars = 100 μm.
