# Supplementary material for: Effect of exercise and diet intervention in NAFLD and NASH via GAB2 methylation
Source: Cell Biosci. 2021 Nov 4;11:189. doi: 10.1186/s13578-021-00701-6 (PMC8569968; doi:10.1186/s13578-021-00701-6)
Supplement: Supplementary file 6 — Additional file 6: Table S2. Clinical traits of NASH mice after intervention. 1Intervention groups: EHFD, exercise plus high fat diet; ELFD, exercise plus low fat diet; HFD, high fat diet; LFD, low fat diet; MCSM, methionine choline sufficient diet (4 weeks). None intervention groups: MCD, methionine choline deficiency diet (8 weeks); MCSC, methionine choline sufficient diet (8 weeks). [file 13578_2021_701_MOESM6_ESM.docx]

Table S2. Clinical traits of NASH mice after intervention.

|  | HFD | EHFD | LFD | ELFD | MCSM | MCD | MCSC |
| --- | --- | --- | --- | --- | --- | --- | --- |
| Weight (g) | 39.11 (2.79) | 35.15 (3.96) | 30.19 (1.52) | 30.00 (1.78) | 29.45 (1.58) | 15.81 (0.75) | 29.70 (1.75) |
| Liver weight (g) | 1.20 (0.15) | 1.15 (0.12) | 1.17 (0.21) | 1.41 (0.17) | 1.13 (0.11) | 0.8 0(0.10) | 1.06 (0.16) |
| Epididymal fat pad (g) | 2.62 (0.49) | 1.55 (0.66) | 0.81 (0.24) | 0.68 (0.14) | 0.91 (0.33) | 0.11 (0.03) | 0.95 (0.30) |
| Triglyceride (mmol/L) | 0.08 (0.02) | 0.12 (0.05) | 0.07 (0.01) | 0.07 (0.03) | 0.08 (0.02) | 0.04 (0.01) | 0.07 (0.01) |
| Cholesterol (mmol/L) | 1.61 (0.38) | 1.03 (0.25) | 1.26 (0.50) | 1.11 (0.38) | 0.73 (0.30) | 0.05 (0.03) | 0.90 (0.24) |
| ALT (IU/L) | 26.25 (4.40) | 27.75 (13.20) | 24.33 (20.60) | 20.57 (13.72) | 26.00 (20.38) | 101.63 (30.08) | 18.25 (15.04) |
| AST (IU/L) | 68.13 (13.45) | 96.38 (37.01) | 72.33 (43.87) | 58.14 (19.02) | 86.13 (35.87) | 144.38 (56.03) | 94.75 (38.02) |

^1^Variables are presented as mean (SD). ALT, alanine aminotransferase; AST, aspartate aminotransferase, NASH, nonalcoholic steatohepatitis. Intervention groups: EHFD, exercise plus high fat die; ELFD, exercise plus low fat diet; HFD, high fat diet; LFD, low fat diet; MCSM, methionine choline sufficient diet (4 weeks); None intervention groups: MCD, methionine choline deficiency diet (8 weeks), MCSC, methionine choline sufficient diet (8 weeks).
